# Sex Differences in Clinical Characteristics and Management of Non-valvular Atrial Fibrillation in a Resource-Limited Setting

**DOI:** 10.7759/cureus.78240

**Published:** 2025-01-30

**Authors:** Frank Jorge Valdez Baez, Gissel Mariana Santana Mejía, Juanico Cedano Ramirez, Catherine Merejo Peña, Cemirame Payan jimenez, Laura Valdez de Leon, Warenny Montero Morillo, Evelina Severino Marte

**Affiliations:** 1 Electrophysiology, Asociacion Instituto Dominicano de Cardiologia, Santo Domingo, DOM

**Keywords:** cha2ds2-va, multivariate analysis, non-valvular atrial fibrillation, oral anticoagulation, resource-limited, sex differences

## Abstract

*Introduction*: Non-valvular atrial fibrillation (NVAF), the most prevalent sustained arrhythmia, significantly increases the risk of complications such as stroke, heart failure, and mortality. Emerging evidence highlights notable sex-related differences in its clinical presentation and management. However, a substantial knowledge gap persists regarding these disparities in resource-limited settings, where data remain scarce and the burden of NVAF is rising.

*Objectives*: This study aims to assess sex-related differences in clinical characteristics, risk factors, and therapeutic management - specifically anticoagulation use, rate control, and rhythm control strategies - among patients with NVAF.

*Methodology*: This observational study included patients diagnosed with NVAF from April 2023 to November 2024. Patients were subsequently stratified by sex to evaluate differences in clinical, demographic, and therapeutic variables. Statistical analyses incorporated univariate, bivariate, and multivariate approaches, with statistical significance set at *P* < 0.05.

*Results*: A total of 594 patients with NVAF were included, of whom 316 (53.2%) were male and 278 (46.8%) were female. Notably, female patients were generally older (77.8 vs. 75.3 years; *P* = 0.006), exhibited higher systolic blood pressure (130.1 vs. 124.2 mmHg; *P* = 0.007), had higher CHA2DS2-VA scores (3.38 vs. 3.1; *P* = 0.008), and displayed a lower prevalence of heart failure with reduced ejection fraction (15.1% vs. 24.4%; *P* = 0.005). In contrast, male patients presented a higher body mass index (BMI 26.2 vs. 24.5 kg/m²; *P* = 0.003) and elevated serum creatinine levels (1.3 vs. 1.09 mg/dL; *P* < 0.001). In the multivariate analysis, an age ≥75 years (odds ratio [OR] = 1.66; *P* = 0.026) and systolic blood pressure (OR = 1.01; *P* = 0.052) were positively associated with female sex, whereas higher BMI (OR = 0.96; *P* = 0.037) and increased serum creatinine levels (OR = 0.32; *P* < 0.001) were inversely associated. These findings underscore distinct differences in risk factors and clinical profiles between the sexes.

*Conclusions*: Female patients frequently present at an advanced age, demonstrate suboptimal blood pressure control, and face an elevated risk of thromboembolism, whereas male patients exhibit a higher BMI and a greater prevalence of heart failure with reduced ejection fraction. These observations underscore the necessity of sex-specific therapeutic strategies.

## Introduction

Non-valvular atrial fibrillation (NVAF), defined as atrial fibrillation (AF) in the absence of significant mitral valvular disease, prosthetic valves, or rheumatic mitral stenosis, is the most common sustained arrhythmia, affecting 59.7 million people globally in 2019, with a prevalence of up to 5.9% in U.S. community-based cohorts. Its burden is rising due to aging populations, hypertension, obesity, and diabetes, particularly in low- and middle-income countries, and is linked to increased risks of stroke, heart failure (HF), dementia, and over 250,000 deaths in 2017. Management focuses on direct oral anticoagulants (DOACs), guided by the CHA2DS2-VASc or CHA2DS2-VA score, with the 2024 guidelines removing female sex as an independent factor in thromboembolic risk [1].

Recent evidence underscores sex-specific differences in AF. In Norway, female patients demonstrated a lower prevalence (0.19% vs. 0.50%) and reduced use of healthcare services (38% vs. 71%) [2]. In Asia, diagnosed female patients were older (72 vs. 67 years; *P* < 0.001) and exhibited higher rates of dyslipidemia (41.7% vs. 36.7%; *P* = 0.002), whereas male patients presented with a higher prevalence of coronary artery disease (21.1% vs. 16.1%; *P* < 0.001) [3]. In Europe, female patients displayed greater endomysial fibrosis, while male patients demonstrated more atrial hypertrophy [4]. In the United States, female patients experienced higher mortality from ischemic strokes (relative risk [RR] 1.17) but a lower incidence of hemorrhages (RR 0.82) [5]. A 2024 meta-analysis demonstrated a higher risk of gastrointestinal bleeding with rivaroxaban in female patients (RR 1.34; *P* < 0.001) [6], and an ablation study reported higher recurrence in this group (hazard ratio [HR] 2.90) [7]. In the Middle East, female patients were prescribed more anticoagulants (84.4% vs. 78.9%; *P* = 0.001) but fewer rhythm control strategies (23.4% vs. 27.3%; *P* = 0.04) [8]. The DECAAF II trial (Delayed Enhancement MRI Determinant of Atrial Fibrillation Catheter Ablation II) revealed that females had higher arrhythmia recurrence (53.3% vs. 40.2%; *P* < 0.01), greater AF burden (21% vs. 16%; *P* < 0.01), smaller reductions in left atrial volume (*P* = 0.019), and worse quality-of-life scores pre- and post-ablation (*P* < 0.01) compared to males, despite similar improvements across sexes. These findings highlight the importance of sex-specific approaches in AF management [9]. Finally, improvements in physical fitness significantly reduced recurrence in this group (HR 0.13 vs. 0.63; *P* = 0.002) [10].

In summary, female patients with NVAF tend to be diagnosed at an older age, experience more comorbidities, have a higher risk of stroke, and encounter reduced access to strategies such as ablation, despite receiving more anticoagulant therapy. Interventions aimed at enhancing physical fitness are especially beneficial in this group.

In low- and middle-income countries such as the Dominican Republic, the high prevalence of comorbidities, limited access to advanced therapies, and scarcity of data underscore the need to analyze differences in the management of NVAF to develop tailored strategies. The Asociación Instituto Dominicano de Cardiología (Dominican Institute of Cardiology Association), as a specialized center dedicated to cardiovascular diseases, primarily provides care to economically disadvantaged patients.

This study aims to evaluate these disparities in a specialized service, examining demographic, clinical, and therapeutic characteristics by sex. This approach aims to optimize care in low-resource settings and addresses a critical gap in the literature regarding sex-related differences in NVAF.

## Materials and methods

This observational study of previously recorded clinical data was conducted at the Asociación Instituto Dominicano de Cardiología, including patients with NVAF who received treatment between April 2023 and November 2024. While this approach provides insights into real-world care patterns in a vulnerable population, it also carries inherent limitations. In particular, relying on existing medical records can result in missing or incomplete data, potentially leading to under- or overestimation of specific outcomes. Additionally, the retrospective nature of data collection limits the degree of control over data quality. These factors should be carefully considered when interpreting the study’s findings.

The principal aim of this study was to analyze sex-related differences in clinical characteristics, risk factors, and therapeutic management - specifically anticoagulation use, rate control, and rhythm control strategies - among patients with NVAF.

The study included outpatient individuals aged 18 years and older who already had a confirmed diagnosis of NVAF before enrollment and were receiving follow-up care in the specialized outpatient clinic at the Asociación Instituto Dominicano de Cardiología.

Patients presenting with valvular AF (significant mitral valvular disease, rheumatic mitral stenosis, or prosthetic valves) - who are managed in the institution’s surgical department - were excluded. Also excluded were patients with recent intracardiac devices, incomplete records, severe comorbidities, or complex arrhythmias that could affect NVAF management. Hospitalized patients, individuals under 18 years of age, and duplicate data from multiple visits during the collection period were likewise not included.

The sample included consecutive patients during the data collection period, with no prior calculation of sample size, thereby minimizing selection bias and including all eligible individuals according to the inclusion criteria. Patients were then classified by sex (male and female) to analyze differences in clinical variables and therapeutic management. A post hoc analysis was conducted to ensure adequate statistical power.

Data were collected using a form specifically designed for this study, structured to facilitate organization and analysis. To ensure data quality, staff received training in implementing these updates, and periodic cross-checks were conducted.

Demographic, clinical, and therapeutic variables were collected to examine sex-related differences in patients with NVAF. Demographic variables included age (<75 and ≥75 years), sex (male or female), insurance type (contributory, with regular access to therapeutic resources, or subsidized, with limited access), and geographic region (urban or rural) to evaluate healthcare access.

Among the clinical variables, systolic blood pressure (SBP) and diastolic blood pressure (DBP) were included, categorized as SBP <140 mmHg and ≥140 mmHg and DBP <90 mmHg and ≥90 mmHg according to international guidelines. BMI was classified as <30 kg/m² (non-obesity) and ≥30 kg/m² (obesity). Hemoglobin (<12 g/dL and ≥12 g/dL), hematocrit (%), and serum creatinine were collected to calculate endogenous creatinine clearance (CrCl) using the Cockcroft-Gault formula, categorized as <50 mL/minute and ≥50 mL/minute. In addition, left ventricular ejection fraction (LVEF) was documented via echocardiography as a continuous variable.

Among the comorbidities and risk factors were hypertension, diabetes mellitus, vascular disease, HF with an LVEF ≤40%, as well as a history of stroke, transient ischemic attack (TIA), hemorrhages (cerebral and gastrointestinal), cancer, anemia, and chronic kidney disease. The use of nonsteroidal anti-inflammatory drugs (NSAIDs) and antiplatelet agents (APAs) was also documented due to their impact on clinical management. Clinical risk was assessed using the CHA2DS2-VA scale, categorized as low-moderate (<3) or high risk (≥3) for thromboembolic events, and the HAS-BLED score, classified as low (<3) or high risk (≥3) for bleeding.

Patients with NVAF were categorized as permanent or non-permanent (paroxysmal, persistent, and long-standing persistent), excluding cases with an unknown classification, using information from their initial diagnosis during the first visit to the department. This observational study utilized the 2020 guideline-based classification of AF, which stratifies episodes by duration and response to rhythm restoration: paroxysmal AF terminates spontaneously or through intervention within seven days; persistent AF continues beyond seven days, often requiring cardioversion; long-standing persistent AF endures for at least 12 months with the possibility of pursuing rhythm control; and permanent AF is accepted by both patient and physician as final, with no further attempts to restore or maintain sinus rhythm, typically based on clinical tolerance and therapeutic decision-making. NVAF was evaluated as symptomatic or asymptomatic, and management strategies included rhythm or rate control, based on data collected during the visit corresponding to the recruitment of data for this registry.

Therapy for the prevention of ischemic events with DOACs, vitamin K antagonists (VKAs), APAs, or none was documented, along with the doses of DOACs used. Other treatments included angiotensin-converting enzyme inhibitors (ACEI), angiotensin receptor blockers (ARBs), angiotensin receptor-neprilysin inhibitors (ARNIs), mineralocorticoids, statins, sodium-glucose cotransporter 2 (SGLT2) inhibitors, beta-blockers, calcium channel blockers, diuretics, digoxin, amiodarone, and propafenone, as well as non-pharmacological interventions such as pacemakers and ablation. Polypharmacy was recorded to evaluate the impact of complex treatment on sex-related differences. For the purposes of this study, polypharmacy was defined as the use of six or more medications.

The statistical analysis compared variables between male and female groups. Univariate analysis was performed to describe the overall characteristics of the sample, with continuous variables presented as means and standard deviations, and categorical variables expressed as frequencies and percentages.

During the bivariate analysis, chi-square (χ²) tests were used for categorical variables, and Fisher’s exact test was employed in cases of low frequencies. Effect size for categorical variables was assessed using Cramér’s V, interpreted as small (≥0.10), moderate (≥0.30), and large (≥0.50). For continuous variables, the student’s t-test was employed for normally distributed data, and the Mann-Whitney test for non-normal distributions. Normality of continuous variables was evaluated using the Kolmogorov-Smirnov test, supplemented with histograms and Q-Q plots to visualize data distribution. Effect size was calculated using Cohen’s d, categorized as small (≥0.20), moderate (≥0.50), and large (≥0.80).

Subsequently, a bivariate logistic regression analysis was performed to evaluate the association between each independent variable and sex. For the multivariate analysis, a logistic regression model was applied to identify independent associations among the selected variables. Candidate variables were those that achieved statistical significance in the bivariate analysis (*P *< 0.05) and those considered clinically relevant. Redundant variables were excluded based on principal component analysis (PCA) and association coefficients (Cramér’s V <0.7 for categorical variables). Collinearity among the selected variables was assessed using the variance inflation factor (VIF), with VIF > 10 indicating significant collinearity. Furthermore, individual variables were prioritized over composite indices, such as CHA2DS2-VA, to avoid both conceptual and statistical duplication.

The final model included only the variables that maintained a *P*-value ≤0.05 in the multivariate analysis, and the results were reported as OR with 95% confidence intervals, ensuring robust statistical and clinical interpretation.

The overall fit of the model was assessed using the -2 log likelihood and the Cox & Snell, Nagelkerke, and McFadden R² coefficients. Its discriminative ability was determined using the receiver operating characteristic (ROC) curve and the area under the curve (AUC). An AUC exceeding 0.7 was considered indicative of acceptable discrimination, whereas values above 0.8 reflected excellent predictive capacity.

Internal validation was performed by splitting the data into training (70%) and testing (30%) sets. The model was trained on the training set and subsequently evaluated on the testing set. Calibration was assessed using the Brier score, calibration curve, and Hosmer-Lemeshow test, with a *P*-value > 0.05 indicating a good fit.

Since the study design did not include a priori sample size calculation, a post hoc statistical power analysis was conducted, establishing a power of 80% and a significance level of α ≤ 0.05. This analysis confirmed that the sample was sufficient to support the findings, taking into account both the effect size and the number of participants to ensure the statistical reliability of the assessed associations. Variables with missing data were examined to identify patterns, and in significant cases, multiple imputation techniques or sensitivity analyses were applied to verify the robustness of the findings and ensure the validity of the conclusions.

Statistical analysis was performed using SPSS software, version 23 (IBM Corp., Armonk, NY).

This study was approved by the Ethics Committee of the Asociación Instituto Dominicano de Cardiología (AIDC-CE-2024-011), implementing strict confidentiality measures through the use of de-identified data to ensure patient privacy. As a retrospective and observational registry, informed consent was not required, in accordance with the ethical standards of the Declaration of Helsinki. No sex-related biases were introduced due to the nature of the study. The authors declare no conflicts of interest and have not received public, private, or commercial funding for either the research or the preparation of the manuscript.

## Results

In this study, a total of 594 patients diagnosed with NVAF were included. Among them, 316 patients (53.2%) were male, whereas 278 patients (46.8%) were female.

The results of Table 1 indicate significant differences between female and male patients with NVAF. The female group demonstrated a higher average age (77.78 ± 10.77 vs. 75.28 ± 11.37 years; *P* = 0.006), elevated SBP (130.08 ± 23.55 mmHg vs. 124.21 ± 22.39 mmHg; *P* = 0.007), and a higher CHA2DS2-VA score (3.38 ± 1.29 vs. 3.1 ± 1.37; *P* = 0.008). Moreover, female patients exhibited a significantly higher LVEF (61.08 ± 14.63% vs. 55.9 ± 17.16%; *P* = 0.025).

**Table 1:** Comparison of clinical and laboratory variables between NVAF patients by sex. The table presents a comparison of means and standard deviations (SDs) for clinical and laboratory variables between male and female patients with non-valvular atrial fibrillation (NVAF). *P*-values were determined using student's t-tests (TS) or Mann-Whitney U tests (MWU), depending on the distribution of the variables. For variables with statistically significant differences (*P* ≤ 0.05), the effect size and statistical power were as follows: age (TS, Cohen’s *d* = 0.23, power = 96.92%), SBP (MWU, *r* = 0.12, power = 89.12%), weight (MWU, *r* = 0.10, power = 21%), BMI (TS, Cohen’s *d* = 0.26, power = 97.57%), creatinine (MWU, *r* = 0.28, power = 96.63%), CHA2DS2-VA (MWU, *r *= 0.11, power = 87.79%), and LVEF (TS, Cohen’s *d* = 0.32, power = 95.30%). SBP, systolic blood pressure; DBP, diastolic blood pressure; BMI, body mass index; CrCl, endogenous creatinine clearance; INR, international normalized ratio; CHA2DS2-VA, congestive heart failure; hypertension; age ≥75 years; diabetes mellitus; stroke or ischemic event; vascular disease; age 65-74 years; HAS-BLED, hypertension, abnormal renal or liver function, history of stroke, bleeding or predisposition, unstable international normalized ratio, age >65 years, drugs/alcohol; LVEF, left ventricular ejection fraction

Variable	Male		Female		P
	n	Mean ± SD	n	Mean ± SD	
Age (years)	316	75.28 ± 11.37	278	77.78 ± 10.77	0.006
SBP (mmHg)	304	124.21 ± 22.39	265	130.08 ± 23.55	0.007
DBP (mmHg)	193	71.97 ± 10.78	165	74.69 ± 12.71	0.145
Weight (kg)	283	74.38 ± 16.68	240	70.7 ± 15.54	0.019
Height (m)	316	1.69 ± 0.12	278	1.71 ± 0.12	0.069
BMI (kg/m²)	283	26.18 ± 6.54	240	24.53 ± 6.28	0.003
Creatinine (mg/dL)	188	1.3 ± 0.49	178	1.09 ± 0.47	<0.001
CrCl (mL/min)	179	57.93 ± 24.68	164	56.66 ± 26.12	0.644
INR	39	2.14 ± 1.36	29	2.68 ± 1.65	0.148
Hemoglobin (g/dL)	44	12.98 ± 1.59	55	12.8 ± 1.17	0.563
Hematocrit (%)	42	39.88 ± 4.84	55	39.44 ± 3.65	0.616
CHA2DS2-VA	316	3.1 ± 1.37	278	3.38 ± 1.29	0.008
HAS-BLED	316	1.42 ± 0.91	278	1.53 ± 0.84	0.059
Polypharmacy	316	2.36 ± 1.73	278	2.37 ± 1.5	0.446
Echocardiogram LVEF (%)	105	55.9 ± 17.16	93	61.08 ± 14.63	0.025

**Figure 1:**
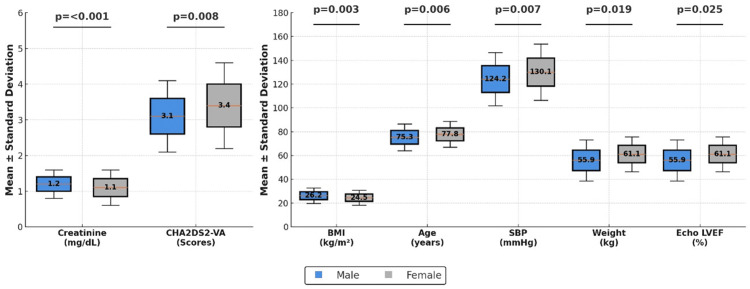
Significant clinical differences by sex in patients with non-valvular atrial fibrillation (NVAF). Box plots comparing significant clinical differences by sex in patients with NVAF. The boxes represent mean values ± standard deviation for each variable, with *P*-values displayed above the boxes indicating the level of statistical significance for differences between sexes. Vertical axis scales were adjusted to improve visualization for variables with smaller ranges, such as creatinine and CHA2DS2-VA. Statistical analyses were conducted using Student’s t-test for normally distributed variables and the Mann-Whitney U test for non-normally distributed variables, with *P* ≤ 0.05 considered significant. SBP, systolic blood pressure (mmHg); BMI, body mass index (kg/m²); CHA2DS2-VA, congestive heart failure, hypertension, age ≥75 years (2 points), diabetes, stroke (2 points), vascular disease, age 65–74 years; Echo LVEF, echocardiogram left ventricular ejection fraction (%); weight (kg)

By contrast, the male group had a higher average weight (74.38 ± 16.68 kg vs. 70.7 ± 15.54 kg; *P* = 0.019) and an increased BMI (26.18 ± 6.54 kg/m² vs. 24.53 ± 6.28 kg/m²; *P* = 0.003). Additionally, male patients demonstrated significantly higher serum creatinine levels (1.3 ± 0.49 mg/dL vs. 1.09 ± 0.47 mg/dL; *P* < 0.001). Figure 1 illustrates the findings presented in Table 1.

No significant differences were observed in DBP, hemoglobin, hematocrit, endogenous creatinine clearance, polypharmacy, or HAS-BLED score between the groups. These findings underscore the importance of considering sex-specific differences in the management and treatment of patients with NVAF, particularly regarding age, blood pressure, thromboembolic risk, and cardiac function.

The results presented in Table 2 compare the distribution of variables between male and female patients with NVAF. A greater proportion of female patients were aged over 75 years (63.67% vs. 51.27%; *P* = 0.002) and exhibited SBP ≥ 140 mmHg (24.91% vs. 15.46%; *P* = 0.005) and DBP ≥90 mmHg (7.27% vs. 1.55%; *P* = 0.007). Furthermore, thromboembolic risk was elevated in this cohort, with a higher percentage of patients presenting elevated CHA2DS2-VA scores (76.62% vs. 67.09%; *P* = 0.01).

**Table 2:** Comparison of categorical clinical and laboratory variables between male and female patients with non-valvular atrial fibrillation (NVAF). The table presents the distribution of categorical variables between male and female patients with NVAF, expressed as absolute frequencies and percentages. Differences were evaluated using the chi-square test (χ²), and for variables with statistical significance (*P* ≤ 0.05), the effect size was calculated with Cramér's V and statistical power was reported. Significant variables included age (Cramér's V = 0.13, power = 90.94%), SBP (mmHg) (Cramér's V = 0.12, power = 89.12%), DBP (mmHg) (Cramér's V = 0.14, power = 87.53%), and CHA2DS2-VA (≥3) (Cramér's V = 0.11, power = 87.79%). SBP, systolic blood pressure; DBP, diastolic blood pressure; BMI, body mass index; CrCl, endogenous creatinine clearance; CHA2DS2-VA, congestive heart failure; hypertension; age ≥75 years; diabetes mellitus; stroke or ischemic event; vascular disease; age 65-74 years; HAS-BLED, hypertension, abnormal renal or liver function, history of stroke, bleeding or predisposition, unstable international normalized ratio, age >65 years, drugs/alcohol; LVEF, left ventricular ejection fraction

Variable	Category	Male, *n* (%)	Female, *n* (%)	Total, *n* (%)	P
Age (years)	<75	154 (48.73%)	101 (36.33%)	255 (42.93%)	0.002
	≥75	162 (51.27%)	177 (63.67%)	339 (57.07%)	
SBP (mmHg)	<140	257 (84.54%)	199 (75.09%)	456 (80.14%)	0.005
	≥140	47 (15.46%)	66 (24.91%)	113 (19.86%)	
DBP (mmHg)	<90	190 (98.45%)	153 (92.73%)	343 (95.81%)	0.007
	≥90	3 (1.55%)	12 (7.27%)	15 (4.19%)	
BMI (kg/m²)	<30	245 (77.53%)	232 (83.45%)	477 (80.30%)	0.070
	≥30	71 (22.47%)	46 (16.55%)	117 (19.70%)	
Hemoglobin (g/dL)	<12	10 (22.73%)	9 (16.36%)	19 (19.19%)	0.424
	≥12	34 (77.27%)	46 (83.64%)	80 (80.81%)	
CrCl (mL/minute)	<50	79 (44.13%)	73 (44.51%)	152 (44.33%)	0.944
	≥50	100 (55.87%)	91 (55.49%)	191 (55.67%)	
CHA2DS2-VA	<3	104 (32.91%)	65 (23.38%)	169 (28.45%)	0.010
	≥3	212 (67.09%)	213 (76.62%)	425 (71.55%)	
HAS-BLED	<3	281 (88.92%)	244 (87.77%)	525 (88.38%)	0.661
	≥3	35 (11.08%)	34 (12.23%)	69 (11.62%)	
Polypharmacy	<6	301 (95.25%)	270 (97.12%)	571 (96.13%)	0.239
	≥6	15 (4.75%)	8 (2.88%)	23 (3.87%)	
Echocardiogram LVEF (%)	≤40	23 (21.9%)	14 (15.05%)	37 (18.69%)	0.217
	>40	82 (78.1%)	79 (84.95%)	161 (81.31%)	

No significant differences were identified in other variables, including BMI, hemoglobin levels, creatinine clearance, HAS-BLED scores, polypharmacy, or LVEF. These findings emphasize the necessity of accounting for sex-related differences in risk stratification and blood pressure management to optimize clinical outcomes in patients with NVAF

Table 3 presents a comparison of categorical variables between male and female patients with NVAF. Significant differences were identified in several comorbidities and treatment modalities. HF or LVEF ≤ 40% was more prevalent in the male group (24.37% vs. 15.11%; *P* = 0.005). Additionally, the use of sodium-glucose cotransporter-2 (SGLT2) inhibitors was significantly higher in the male group (10.13% vs. 5.4%, *P* = 0.048).

**Table 3:** Distribution of medical insurance, geographic origin, comorbidities, and therapies by sex in patients with non-valvular atrial fibrillation (NVAF). The table presents the distribution of categorical variables between male and female patients, expressed as absolute frequencies and percentages. Differences were evaluated using the chi-square test (χ²), and the effect size calculated with Cramér's V is reported. For variables with statistical significance (*P* ≤ 0.05), statistical power is included. Significant variables included HF/LVEF ≤ 40% (Cramér's V = 0.12, power = 89.53%) and the use of SGLT2 inhibitors (Cramér's V = 0.11, power = 81.58%). HF/LVEF ≤ 40%, heart failure/left ventricular ejection fraction ≤ 40%; TIA, transient ischemic attack; NSAIDs, nonsteroidal anti-inflammatory drugs; APAs, antiplatelet agents; ACEIs, angiotensin-converting enzyme inhibitors; ARBs, angiotensin II receptor blockers; ARNIs, neprilysin and angiotensin receptor inhibitors; HCTZ, hydrochlorothiazide; SGLT2, sodium-glucose cotransporter-2 inhibitors

Variable	Male, *n* = 316, *n* (%)	Female, *n* = 278, *n* (%)	Total, *n* = 594, *n* (%)	P
Medical insurance				
Subsidized	281 (88.93%)	255 (91.73%)	536 (90.23%)	0.504
Contributory	33 (10.44%)	22 (7.91%)	55 (9.26%)	
None	2 (0.63%)	1 (0.36%)	3 (0.51%)	
Geographic origin				
Urban	267 (84.49%)	233 (83.81%)	500 (84.18%)	0.821
Rural	49 (15.51%)	45 (16.19%)	94 (15.82%)	
Comorbidities				
Hypertension	287 (90.82%)	259 (93.17%)	546 (91.92%)	0.296
HF/LVEF ≤ 40%	77 (24.37%)	42 (15.11%)	119 (20.03%)	0.005
Diabetes mellitus	64 (20.25%)	59 (21.22%)	123 (20.72%)	0.771
Stroke, TIA, cerebral hemorrhage	59 (18.67%)	55 (19.78%)	114 (19.19%)	0.731
Vascular disease	35 (11.08%)	22 (7.91%)	57 (9.59%)	0.192
Gastrointestinal bleeding	25 (7.91%)	25 (8.99%)	50 (8.42%)	0.636
Cancer	14 (4.43%)	6 (2.16%)	20 (3.37%)	0.126
Chronic kidney disease	11 (3.48%)	12 (4.32%)	23 (3.87%)	0.754
Anemia	10 (3.16%)	9 (3.24%)	19 (3.20%)	0.960
NSAIDs/APAs	7 (2.22%)	4 (1.44%)	11 (1.85%)	0.484
Therapies				
Pacemaker	195 (61.71%)	163 (58.63%)	358 (60.27%)	0.496
ACEI/ARB/ARNI	194 (61.39%)	184 (66.19%)	378 (63.64%)	0.260
Beta-blockers	158 (50.00%)	153 (55.04%)	311 (52.36%)	0.253
Furosemide-HCTZ	117 (37.03%)	108 (38.85%)	225 (37.88%)	0.710
Calcium channel blocker	71 (22.47%)	61 (21.94%)	132 (22.22%)	0.956
Mineralocorticoids	63 (19.94%)	48 (17.27%)	111 (18.69%)	0.467
Amiodarone	32 (10.13%)	33 (11.87%)	65 (10.95%)	0.584
Statins	32 (10.13%)	26 (9.35%)	58 (9.76%)	0.858
SGLT2 inhibitors	32 (10.13%)	15 (5.40%)	47 (7.91%)	0.048
Digoxin	24 (7.59%)	16 (5.76%)	40 (6.73%)	0.466
APAs	21 (6.65%)	14 (5.04%)	35 (5.89%)	0.511
Ablation	8 (2.53%)	6 (2.16%)	14 (2.36%)	0.977
Propafenone	2 (0.63%)	1 (0.36%)	3 (0.51%)	1.000

No significant differences were observed between the groups regarding variables such as type of medical insurance, geographic origin, comorbidities including hypertension, diabetes mellitus, or a history of cerebrovascular events or cerebral hemorrhages, nor in the use of common therapies such as beta-blockers, calcium channel blockers, digoxin, amiodarone, ablation, or antiplatelet agents. These results indicate a consistency in therapeutic management and clinical characteristics between male and female patients.

These findings suggest that, although significant differences exist in certain comorbidities and specific treatments, other variables related to health management and medication exhibit a similar distribution between male and female groups. The noteworthy differences observed in SGLT2 inhibitor use and the higher prevalence of HF in male patients may have implications for therapeutic approaches and the personalized management of NVAF based on sex. However, the variables without statistical significance support the notion that, in some aspects, patients of both sexes can be treated similarly.

In Table 4, variables pertaining to the classification of AF, symptomatology, management strategies, and anticoagulant therapies were compared between male and female patients with NVAF. Findings indicated that the permanent form of AF was the most prevalent in both sexes, accompanied by a high proportion of asymptomatic patients. Concerning management strategies, rate control predominated over rhythm control, while DOACs, particularly apixaban, were the most commonly utilized therapy in both groups.

**Table 4:** Comparison of classification, symptomatology, management strategies, and anticoagulant therapies by sex in patients with non-valvular atrial fibrillation. The table illustrates the distribution of variables related to the classification of atrial fibrillation, symptomatology, management strategies, and anticoagulant therapies in male and female patients. Data are presented as absolute frequencies and percentages. Differences between sexes were evaluated using the chi-square test (χ²), applying a significance level of *P* ≤ 0.05. Doses are quantified in milligrams (mg) and frequencies in hours. APAs, antiplatelet agents; OAC, oral anticoagulant; DOAC, direct oral anticoagulant; VKA, vitamin K antagonist

Variable	Category	Male, *n* (%)	Female, *n* (%)	Total, *n* (%)	P
Classification	Permanent	194 (65.76%)	169 (63.30%)	363 (64.59%)	0.541
	Non-permanent	101 (34.24%)	98 (36.70%)	199 (35.41%)	
Symptomatology	No	293 (92.72%)	250 (89.93%)	543 (91.41%)	0.225
	Yes	23 (7.28%)	28 (10.07%)	51 (8.59%)	
Strategy	Rate control	284 (89.87%)	247 (88.85%)	531 (89.40%)	0.686
	Rhythm control	32 (10.13%)	31 (11.15%)	63 (10.60%)	
Therapy	Anticoagulants	274 (86.71%)	250 (89.93%)	524 (88.22%)	0.612
	None	27 (8.54%)	17 (6.11%)	44 (7.41%)	
	APAs	11 (3.48%)	9 (3.24%)	20 (3.37%)	
	OAC plus APAs	4 (1.27%)	2 (0.72%)	6 (1.00%)	
Type OAC	DOAC	238 (85.61%)	220 (87.30%)	458 (86.42%)	0.238
	VKA	40 (14.39%)	32 (12.70%)	72 (13.58%)	
Type DOAC	Apixaban	154 (64.71%)	153 (69.55%)	307 (67.03%)	0.368
	Rivaroxaban	79 (33.19%)	64 (29.09%)	143 (31.22%)	
	Edoxaban	5 (2.10%)	2 (0.91%)	7 (1.53%)	
	Dabigatrán	0 (0.00%)	1 (0.45%)	1 (0.22%)	
Dose DOAC	Apixaban 5/12 hours	106 (44.53%)	86 (39.10%)	192 (41.92%)	0.113
	Apixaban 2.5/12 hours	47 (19.75%)	66 (30.00%)	113 (24.67%)	
	Rivaroxaban 20/24 hours	47 (19.75%)	29 (13.18%)	76 (16.59%)	
	Rivaroxaban 15/24 hours	32 (13.45%)	35 (15.91%)	67 (14.63%)	
	Edoxaban 30/24 hours	4 (1.68%)	2 (0.91%)	6 (1.31%)	
	Rivaroxaban 10/24 hours	1 (0.42%)	1 (0.45%)	2 (0.44%)	
	Edoxaban 60/24 hours	1 (0.42%)	0 (0.00%)	1 (0.22%)	
	Dabigatrán 150/12 hours	0 (0.00%)	1 (0.45%)	1 (0.22%)	

No statistically significant differences were observed between sexes in any of the evaluated variables, suggesting a high degree of homogeneity in clinical characteristics and therapeutic strategies.

Table 5 presents the results of the bivariate and multivariate logistic regression analyses aimed at identifying factors associated with female sex in patients with NVAF.

**Table 5:** Results of bivariate and multivariate logistic regression analyses to identify factors associated with female sex in patients with non-valvular atrial fibrillation (NVAF). The table presents the outcomes of bivariate and multivariate logistic regression analyses conducted to identify factors associated with female sex in patients with NVAF. Reported are *P*-values, OR, and 95% confidence intervals (95% CI). Associations were evaluated using the logistic regression model, applying a significance level of *P* ≤ 0.05. In the multivariate model, variables were simultaneously adjusted to identify independent predictors. SBP, systolic blood pressure; DBP, diastolic blood pressure; BMI, body mass index; CHA2DS2-VA, congestive heart failure, hypertension, age ≥75 years, diabetes mellitus, prior stroke, vascular disease, age 65-74 years; HF/LVEF, heart failure/left ventricular ejection fraction; SGLT2, sodium-glucose cotransporter-2 inhibitors

Bivariate logistic regression	Odds ratio (OR)	95% CI	P
Age (years)	1.02	1.01-1.04	0.007
SBP (mmHg)	1.01	1.00-1.02	0.003
Weight (kg)	0.99	0.97-1.00	0.011
BMI (kg/m²)	0.96	0.93-0.99	0.004
Creatinine (mg/dL)	0.36	0.22-0.60	<0.001
CHA2DS2-VA	1.18	1.04-1.33	0.010
Echocardiogram LVEF (%)	1.02	1.00-1.04	0.026
Age ≥ 75 years	1.67	1.20-2.32	0.002
SBP ≥ 140 (mmHg)	1.78	1.18-2.70	0.006
DBP ≥ 90 (mmHg)	4.71	1.31-16.86	0.017
CHA2DS2-VA ≥3	1.61	1.12-2.31	0.010
HF/LVEF ≤ 40%	0.55	0.36-0.84	0.005
SGLT2 inhibitors	0.51	0.27-0.96	0.036
Multivariate logistic regression			
SBP (mmHg)	1.01	1-1.02	0.052
Creatinine (mg/dL)	0.32	0.18-0.56	<0.001
Age ≥ 75 years	1.6	1-2.54	0.048
BMI (kg/m²)	0.96	0.92-1	0.037

In the bivariate analysis, several variables were found to be significantly associated with female sex. Age (*P* = 0.007, OR = 1.02) demonstrated a positive relationship, indicating that as age increases, so does the likelihood of being female. SBP ≥ 140 mmHg (*P* = 0.006, OR = 1.78) was also positively associated with female sex, suggesting that patients with higher systolic hypertension are more likely to belong to this group. Additionally, BMI (*P* = 0.004, OR = 0.96) was negatively correlated, implying that lower BMI may be a predictive factor for female sex. Creatinine (*P* < 0.001, OR = 0.36) was also significant, with lower creatinine levels linked to a higher likelihood of being female. HF or LVEF ≤ 40% (*P* = 0.005, OR = 0.55) exhibited an inverse relationship with female sex, indicating that female patients with NVAF were less likely to have HF with reduced ejection fraction. CHA2DS2-VA (*P* = 0.01, OR = 1.18) and LVEF (*P* = 0.026, OR = 1.02) showed a positive relationship with female sex. Finally, DBP ≥ 90 mmHg (*P* = 0.017, OR = 4.71) was also significant, with an increased risk of being female in patients with elevated DBP.

In the multivariate logistic regression analysis, an increase of 1 mmHg in SBP was associated with a 1% increase in the probability of being female (OR = 1.01, 95% CI, 1.00-1.02; *P* = 0.052), while higher creatinine levels showed an inverse association, reducing this probability by 68% for each 1 mg/dL increase (OR = 0.32, 95% CI, 0.18-0.56; *P* < 0.001). Being over 75 years old increased the probability by 60% (OR = 1.60, 95% CI, 1.00-2.54; *P* = 0.048), and for each additional unit of BMI, the probability decreased by 4% (OR = 0.96, 95% CI, 0.92-1.00; *P* = 0.037).

The multivariate model exhibited an acceptable fit in the total dataset, with a Nagelkerke R² of 0.13 and a McFadden R² of 0.07, along with a moderate discriminative ability (AUC = 0.692). In internal validation using an independent test set (30% of the data), the AUC was 0.632, indicating a moderate discriminative capacity for predicting female sex. Model calibration was assessed using the Brier Score (0.249), which reflected good accuracy of predicted probabilities, and the Hosmer-Lemeshow test (statistic = 17.26, *P* = 0.045), which indicated a moderate fit. The calibration curve demonstrated that predicted probabilities were reasonably aligned with observed probabilities.

Based on the values of the variables included in the final model, a prediction was constructed using the obtained coefficients. It was found that for a combination of SBP (1.01), creatinine (0.32), age ≥75 years (1.60), and BMI (0.96), the estimated probability of being female was 0.75 (75%), with odds of 2.97, indicating that the probability of being female is approximately three times higher in the presence of these factors. Low creatinine levels and age ≥75 years emerged as the most influential predictors in the model.

## Discussion

This study on patients with NVAF indicates that the female group tends to be older, exhibits poorer control of SBP, and faces a higher thromboembolic risk, highlighting the need for more personalized management. In contrast, the male group demonstrates a higher BMI and creatinine levels, suggesting a greater metabolic and renal burden. Although the type of anticoagulant therapy does not differ significantly, differences in comorbidities and risk factors underscore the importance of considering sex to optimize treatment and improve clinical outcomes, emphasizing the need for further research. These differences are supported by published literature.

The older age observed among female patients may reflect delays associated with less apparent symptoms and complex risk factors. Previous studies highlight higher rates of diagnosis in emergency settings and reduced cardiological follow-up in female patients compared to their male counterparts [11].

Blood pressure control in the female group with NVAF is complex, influenced by a higher prevalence of hypertension, particularly in the postmenopausal stage [12], and the influence of the renin-angiotensin-aldosterone system [13]. Comorbidities such as diabetes and chronic kidney disease, which increase with age, further exacerbate the risk of arrhythmia and complicate blood pressure control. A study conducted in Canada reported that female patients with AF are more likely to have higher SBP levels [11]. Blood pressure, a critical component of the CHA2DS2-VA score, has also been identified as a factor suboptimally controlled in female patients with NVAF, thereby contributing to a higher risk of cardiovascular events. In the SPRINT (Systolic Blood Pressure Intervention Trial) study, intensive blood pressure control reduces the risk of AF, although no specific differences in treatment efficacy have been observed between males and females [14,15].

Female patients with NVAF often exhibit a higher prevalence of comorbidities such as hypertension, diabetes, and chronic kidney disease, which further increase thromboembolic risk through endothelial dysfunction and coagulation alterations. Studies indicate higher CHA2DS2-VA scores in female patients, primarily attributable to advanced age, hypertension, and a history of cerebrovascular accidents [11,16]. Furthermore, the LIFE (Losartan Intervention For Endpoint Reduction in Hypertension) study demonstrated a higher risk of stroke in hypertensive patients older than 64 years, even after adjusting for other factors [6]. This risk, widely documented, remains an independent marker in the CHA2DS2-VA model [17,18].

In this study, male patients demonstrated a higher BMI, elevated creatinine levels, and a greater prevalence of HF. On the one hand, obesity contributes to conditions such as hypertension and diabetes, which facilitate the onset of AF [19]. On the other hand, the coexistence of AF and HF is common, with each condition potentially exacerbating the other. AF can deteriorate cardiac function by inducing a rapid and irregular ventricular response, while HF can create a favorable environment for the development of AF due to pressure and volume overload in the atria [20]. Male patients exhibit a higher incidence of HF at younger ages compared to female patients. However, due to greater post-diagnosis survival in the latter group, the overall prevalence of HF tends to equalize between both sexes in older ages. It is important to note that female patients typically develop HF at an older age and more frequently present with diastolic dysfunction, whereas male patients tend to develop HF with systolic dysfunction [21]. The DAPA-HF (Dapagliflozin And Prevention of Adverse outcomes in Heart Failure) and EMPEROR-Preserved (Empagliflozin Outcome Trial in Patients with Chronic Heart Failure with Preserved Ejection Fraction) studies analyzed the relationship between HF, AF, and the use of SGLT2 inhibitors. These studies confirmed that male patients have a higher prevalence of HF with reduced ejection fraction and greatly benefit from the use of SGLT2 inhibitors, such as dapagliflozin and empagliflozin [22-24].

Obesity is a significant risk factor for the development of AF. Studies have demonstrated that excess adipose tissue, particularly epicardial adipose tissue, can contribute to the structural and electrical remodeling of the heart, thereby increasing susceptibility to arrhythmias such as AF [25]. Furthermore, obesity not only elevates the risk of developing HF but also influences its progression and prognosis. The concept of the *obesity paradox* suggests that, in certain cases, obese patients with HF may have a better prognosis than those with normal weight; however, this phenomenon remains a subject of debate and ongoing study [26]. In male patients, the observed differences are also corroborated by the literature. The VITAL Rhythm (Vitamin D and Omega-3 Trial Rhythm Study) highlighted that BMI is significantly higher in this group, which could partially explain the differences in AF prevalence [27].

The relationship of male sex with HF, renal dysfunction, and AF was investigated in the CARDIOREN (Cardiorenal Spanish Registry) study, demonstrating that these patients with cardiorenal disease had higher probabilities of developing HF with reduced ejection fraction (HFrEF) (OR, 3.13; *P* < 0.005), ischemic cardiomyopathy (OR, 2.17; *P* = 0.003), hypertension (OR, 2.11; *P* = 0.009), AF (OR, 1.71; *P* = 0.025), and hyperkalemia (OR, 2.43; *P* = 0.005) [28]. Obesity contributes to hypertension and diabetes, which, in turn, affect renal and cardiac function, thereby increasing the risk of AF. The presence of AF can worsen HF and renal dysfunction, creating a cycle of progressive deterioration.

In the context of low-income countries, our findings on sex differences in NVAF are consistent with the results of the RE-LY AF registry (Randomized Evaluation of Long-Term Anticoagulant Therapy), which revealed that female patients in these regions receive fewer rhythm control strategies (42.3% versus 50.3% in males; OR, 0.69; *P*<0.001) and face a higher risk of stroke (OR, 1.59; *P *= 0.005), despite a similar rate of anticoagulation (67.0% versus 67.8%, OR, 0.90; *P* not significant) [29]. In our study, we also identified that female patients presented with older age, higher SBP, and a higher CHA2DS2-VA score, reflecting a comorbidity burden similar to that described in RE-LY AF. These similarities underscore the need to improve access to advanced therapies and risk control strategies in this group, particularly in resource-limited settings such as the Dominican Republic.

The differences identified in this study are supported by robust statistical analysis, showing power exceeding 95% for quantitative variables such as BMI, age, and creatinine, and approaching 90% for qualitative variables such as age range (≥75 years) and SBP control (≥140 mmHg).

The multivariate model was designed to minimize redundancy and focus on clinically relevant variables. PCA identified high correlations between weight and BMI (*r *= 0.81), leading to the inclusion of BMI due to its greater clinical relevance in adjusting weight for height. Similarly, age was prioritized over CHA2DS2-VA to avoid redundancy, with the age range ≥75 years selected as it highlights a high-risk subgroup, particularly among females, supported by prior evidence from NVAF guidelines. These choices simplified the model while maintaining clinical relevance.

Independent variables like BMI (kg/m²), SBP (mmHg), and creatinine (mg/dL) were included based on their significance and low collinearity, confirmed through VIF (≤1.02) and tolerance values (0.98-0.99). Excluding categorical variables such as SBP ≥ 140 mmHg and CHA2DS2-VA ≥ 3, despite their statistical significance in bivariate analysis, was justified by their conceptual redundancy with included variables. This approach optimized the model’s parsimony, improving its interpretability and generalizability to clinical practice.

The model demonstrated modest explanatory power (Nagelkerke R² = 0.13, McFadden R² = 0.07) and moderate discriminative ability (AUC = 0.692 in the total dataset, AUC = 0.632 in internal validation). These metrics suggest the model is a practical tool for identifying sex-related differences in NVAF, particularly in resource-limited settings where targeted interventions are essential. The Brier Score (0.249) indicates good prediction accuracy, though the Hosmer-Lemeshow test (*P* = 0.045) reflects moderate calibration, suggesting potential for further refinement.

These findings reinforce the model's utility for guiding clinical decisions, particularly in prioritizing high-risk groups such as older females with NVAF. By balancing statistical precision and clinical relevance, the model supports resource allocation and treatment planning in diverse and resource-constrained environments. Future research should focus on validating the model in external datasets and exploring additional predictors to enhance its performance.

A key finding of our study is the high prevalence of permanent AF within our cohort, primarily attributed to the demographic profile of our patients, characterized by a significant proportion of asymptomatic, pacemaker-dependent, and elderly individuals. These factors often lead clinicians to favor rate-control strategies over rhythm control, as minimal symptoms and pacemaker use facilitate stricter rate management. Additionally, the temporal gap between initial AF stratification and the documentation of therapeutic strategies during the final recruitment visit limits the capture of prior interventions, potentially underrepresenting the use of rhythm control and contributing to the observed high percentage of permanent AF in non-permanent NVAF patients.

The low ablation rate in our cohort can be attributed to several factors. The predominance of elderly, pacemaker-dependent, and asymptomatic patients favored rate-control strategies over rhythm control. Additionally, retrospective and incomplete HF data limited the assessment of its relationship with AF stratification and therapeutic decisions. The lack of consistent left atrial size measurements further restricted individualized treatment approaches. Logistical barriers and costs also influenced the preference for rate control, particularly in stable and asymptomatic patients.

Limitations

This study presents limitations inherent to its retrospective and cross-sectional design, which complicates the establishment of causal relationships and makes it susceptible to selection and temporal biases. Additionally, the data were collected from a single specialized center, limiting its representativeness for the general population with NVAF, particularly in community settings or primary care levels. The sample size was not calculated a priori, although post hoc analysis indicated adequate statistical power. However, this may have limited the ability to detect significant differences in specific subgroups. Furthermore, the quality of the data depended on preexisting medical records, introducing the risk of errors, missing data, and variability in the definitions of clinical variables.

Furthermore, echocardiographic measurements of the left atrium, such as diameter, area, and volume, were not included due to the high variability in available reports, which made it difficult to obtain homogeneous and analyzable data. Psychosocial, economic, or cultural factors that could influence the observed sex differences were also not considered. The lack of longitudinal follow-up limited the assessment of the prognostic impact of these differences, and the absence of comparisons with other populations or geographic contexts restricted the generalizability of the findings, even in low-income countries. Despite these limitations, the results provide relevant information on sex differences in NVAF and underscore the need for additional studies with prospective designs and longitudinal follow-up to validate and expand these findings.

Despite these limitations, the observational study design was selected for its capacity to efficiently and systematically analyze previously recorded clinical data. This approach is particularly advantageous in resource-limited settings such as the Dominican Republic, as it allows for the optimal utilization of available medical records, reduces the costs associated with prospective studies, and provides valuable insights into historical care patterns within a vulnerable population.

The findings of this study support the development of clinical guidelines and public health policies tailored to resource-limited contexts, aiming to improve the equitable and effective management of NVAF. A sex-differentiated approach is proposed: in female patients, strict blood pressure control and thromboembolic risk prevention should be prioritized; in male patients, early diagnosis and management of HF and obesity are recommended. To enhance treatment outcomes, strategies should include patient stratification for early rhythm control, enabling the identification of individuals who may benefit most from rhythm-control interventions. Additionally, it is essential to implement early detection programs, ensure equitable access to anticoagulants through subsidies, train healthcare personnel on sex-specific management differences, and strengthen primary care services. These measures collectively aim to promote a comprehensive and timely approach to NVAF care, particularly in vulnerable populations within resource-limited settings.

## Conclusions

This study demonstrated that female patients with NVAF are generally older, exhibit suboptimal blood pressure control, and experience a higher thromboembolic risk, whereas male patients display a higher body mass index, increased creatinine levels, and a greater prevalence of HF with reduced ejection fraction. These observations highlight notable differences in clinical characteristics and therapeutic approaches.
